# Hospital Festivals and Meetings

**Published:** 1894-06-30

**Authors:** 


### HOSPITAL FESTIVALS AND MEETINGS.

### CHELSEA HOSPITAL FOR WOMEN.

The festival dinner of the Chelsea Hospital for Women was held on Tuesday, June 26th, at the Hotel Metropole, Sir Algernon Borthwick, a member of the Board of Management, presiding.

Sir A. Borthwick gave the toast of "The Queen." Assembled as they were, he said, in the name of charity, the first thought to come to their hearts must be a wish to drink the health of her whom he might call the Queen of Charity, whose heart beat responsive to every woe, whether that of the entombed miners of whose fearful disaster they were all aware, or that of the widow of the brave man who had inherited a grand name and had had the honour of being chosen the first man of the French Republic. It was a great disaster that had fallen upon that nation. Far be the day from us when such a misfortune should occur in this country, but in this age, when Anarchists dared the most infamous crimes, they must be prepared to impress upon the people the necessity of rallying round the Throne, which was the emblem or order, and round the sceptre of the Queen; and they must be resolved to maintain the old constitution of the empire, and to preserve her Gracious Majesty with their arms as she had won their hearts.

Sir A. Borthwick, in proposing "The Prince and Princess of Wales and the rest of the Royal Family," said the toast had a special claim upon them, in that the Royal Family were directly interested in their Hospital, and he hoped that on another occasion some scion of the Royal House would occupy the chair he now filled. Their principal patron was the Princess of Wales, and the Princess Christian, the Duchess of Albany, the Princess Frederica, and the Duchess of Teck were also patronesses. All and every one of them were known for their good work and constant attention to all matters of health and surgical treatment. In giving the toast, he trusted they would not forget the last added member of that family. He had not yet attained much more than a jubilee of hours, but he would have to put on seven-league boots early in life if he wished to tread in the footsteps of his father, his grandfather, and his great-grandfather—and be as active as his sires in the propagation of good works and in taking all possible opportunities of furthering the public advantage in the vast realms over which in the fulness of time—years hence, he hoped—that young Prince would be called to rule. Let them rejoice in the strength of the Royal Family, and drink with all their hearts to the four generations by which it was now represented.

The Chairman then proposed the toast of the evening, "Prosperity to the Chelsea Hospital for Women." He was glad, he said, to see ladies present, their best qualities were always shown to those in pain and suffering. Their hospital was founded to assist the physically weaker sex, and no appeal to charity could be based upon any sentiment more tender. The hospital itself had been passing through a time of pain and suffering—certain accusations against it, which had been perhaps too freely ventilated, had necessitated the appointment of a committee of inquiry. The pronouncement of that committee had not yet been delivered; but he believed that with regard to the earlier charges, relating to the drainage and the scarlet fever cases, it had been proved that the hospital did its duty as well and swiftly as possible. A fair inquiry had been courted, and they would shortly know the result; but this much he could say, that that result would not be prejudicial to the interests of the hospital, but would encourage them to go on and prosper. It would also prove useful by stirring up to measures of reform, and the first to adopt such measures, he was sure, would be the gentlemen whose names had been slurred—in most cases most unjustly slurred. The hospital especially needed friends just now, and the best way to tsetify faith in the institution was to subscribe liberally to it. This was a crisis in the history of the institution. The breath of scandal was capable of doing great mischief, but in this instance it was a mischief that would be brushed away. He called upon them to empty their glasses and their purses.

In the absence of Mr. Jonathan Hutchinson, this toast was responded to by Dr. Robert Barnes, honorary consulting physician. Other toasts were "The Treasurer," "The Guests," and "The Chairman." During the evening the treasurer, Mr. Henry Wright, announced contributions to the amount of £1,728 11s.

---

### UNIVERSITY COLLEGE HOSPITAL.

The festival dinner in aid of the funds of the North London, or University College, Hospital, was held at the Criterion Restaurant, Piccadilly, on Wednesday, June 13th. The Hon. Mr. Justice Charles was in the chair. There were present: The Hon. L. W. Rothschild, Sir Douglas Galton (treasurer), Professor J. Russell-Reynolds, Mr. B. L. Cohen, M.P., and others. In proposing "Prosperity to the Hospital," the Chairman said the institution first opened its doors sixty years ago, having commenced its career as a dispensary in 1828. Its work, Mr. Justice Charles remarked, had been almost unexampled for magnitude and usefulness, but now more room was necessary and the institution required reconstruction. Mrs. Nathaniel Montifiore had already founded a bed and a cot, and now she had promised to endow another in memory of her father. During the whole of its existence the hospital had been in a chronic condition of impecuniosity; they were always in need of money, and never worse off than at present. The hospital was totally inadequate for the purposes it was intended to serve, the population around having increased so enormously, the chairman pointed out. Money was being collected to reconstruct the building.

**HOSPITAL FESTIVALS AND MEETINGS**—*continued.*

Last year the in-patients numbered 3,228, and the out-patients 49,486, showing a large increase upon the previous year. The legacies and donations had somewhat fallen off, and were less by £801 than in 1892. The hospital was in great financial need, and appeals for generous help were being urged. At the conclusion of Mr. Justice Charles' speech, and others, the Secretary announced that the donations had reached a total of £4,540

### SAMARITAN FREE HOSPITAL FOR WOMEN AND CHILDREN.

The annual festival dinner of this charity was celebrated in the Whitehall Rooms of the Hotel Metropole the second week in June, the Duke of Fife, K.T., presiding. His Grace, in giving "Prosperity to the Samaritan Free Hospital," observed that the medical tendency of the age was towards specialism, and the sad cases for which this hospital existed had a special claim to be treated apart from all others. The Samaritan Hospital, he reminded those present, had been associated with the career and the medical victories of a great English surgeon, one who within its walls had taught the world to perform with reasonable security and safety an operation which at one time was considered fatal. His genius and special studies had revolutionised the treatment of those terrible diseases of women which at one time were regarded as incurable. (Applause.) His Grace referred to the name of Sir Spencer Wells. Since this institution was founded 50 years ago it had grown from just a few rooms in a dwelling house to the large and commodious building in Marylebone Road. During the half century of its existence 315,000 women and poor children had been treated within its walls entirely without payment, and of that number nearly 13,000 were in-patients. Patients were received from all parts of the United Kingdom, and even from abroad. At the present time a West Indian woman had come from her home to be treated. The hospital depended entirely upon voluntary contributions. It has no endowment whatever, and it required £6,000 a year for maintenance. He understood, the chairman further explained, that fears were entertained that the institution would have to diminish its means of relieving suffering and prolonging life unless larger funds were forthcoming, but he was sure they would not allow such a state of things to come about, for the hospital brought the triumphs of science to the bedside of the poor and helpless, and conferred incalculable benefits upon generations yet unborn.

Lord Leigh, president of the institution, responded to the Duke's speech. The other toasts submitted were "The Medical Officers," proposed in a sympathetic speech by Mr. Diggle, and ably responded to by Mr. Knowsley Thornton; "The Ladies," proposed by Sir Charles Hall, M.P., and "The Chairman, His Grace the Duke of Fife, K.T.," all of which had been preceded by toasts responded to in loyal terms to her most Gracious Majesty the Queen and other members of the Royal Family.

The banquet was rendered exceedingly bright by the presence of a large number of ladies. Others present were Lord Leigh (president), His Excellency the Portuguese Minister, Lord Windsor, the Hon. Mrs. Knowsley Thornton, Sir Spencer Wells, Sir Charles Hall, M.P., Sir Horace Farquhar. the Hon. and Rev. Canon Leigh, Canon Barker, and many others.

The festival dinner proved also a great pecuniary success, subscriptions amounting to over £2,000 being announced by Mr. Scudamore, the energetic secretary, at the close of the proceedings.

